# Developing a New Instrument for Assessing Acceptance of Change

**DOI:** 10.3389/fpsyg.2016.00802

**Published:** 2016-05-30

**Authors:** Annamaria Di Fabio, Alessio Gori

**Affiliations:** Department of Education and Psychology (Psychology Section), University of FlorenceFlorence, Italy

**Keywords:** acceptance of change, resistance to change, acceptance of change scale, psychometric properties, positive psychology

## Abstract

This article focuses on the usefulness of going beyond the concept of resistance to change and capitalizing on the use of a model that includes positivity and acceptance of change. We first discuss the theoretical background of this new construct in the work and organizational fields and then evaluate the psychometric properties of a new measure for assessing acceptance of change. The results of exploratory factor analysis indicated a factor structure with five principal dimensions; besides confirmatory factor analysis (CFA) goodness of fit indices indicated a good fit of the model to the data. All the dimensions showed good values of internal consistency. The results of the present study indicate that the Acceptance of Change Scale (ACS) is a brief and easily administered instrument with good psychometric properties that can promote the development of clients' strengths and the growth of a sense of Self, thereby helping them choose their own way without losing any opportunities in their lives and their work.

## Introduction

In modern societies, a person's ability to face change is highly valued at the individual level, in the world of work, and in society.

At the individual level, understanding and accepting change is crucial for personal development, particularly because coping with change is often very difficult for many individuals (Wanberg and Banas, 2000).

In today's rapidly changing world of work, people need to deal with change constructively as a good attitude to change can help them find ways of meeting challenges successfully thereby promoting their well-being (Van den Heuvel et al., 2013; Di Fabio et al., 2014; Di Fabio and Palazzeschi, 2015a). Indeed, at the societal level, people who accept change positively can more easily respond to the demands of modern societies. Some people view change as an opportunity to grow and learn with positive implications for their personal development, for creating readiness for change in them (e.g., Armenakis et al., 1993); for “successful planned change” (Miller et al., 1994) in the workplace; for promoting relational management (Di Fabio, 2015b; Di Fabio and Kenny, 2016), well-being (Di Fabio and Bucci, 2015), and psychological strengths (Di Fabio, 2011; Di Fabio and Kenny, 2012a,b; Di Fabio and Palazzeschi, 2012, 2015a; Di Fabio and Saklofske, 2014a,b; Di Fabio and Kenny, 2015); and for improving their relational civility (Di Fabio and Gori, in review).

Several researchers have identified factors that may be associated with people's openness to change (Miller et al., 1994; Judge et al., 1999; Wanberg and Banas, 2000) or resistance to change (Oreg, 2003), but little empirical work has been done in this area, especially as regards acceptance of change. In organizational psychology in particular, a body of literature has grown on resistance to change (Dent and Goldberg, 1999; Folger and Skarlicki, 1999; Oreg, 2003, 2006; Ford et al., 2008; van Dam et al., 2008), and most of the reported studies subscribe to the definition of resistance to change as a “restraining force moving the direction of maintaining the status quo” (Lewin, 1952; Piderit, 2000, p. 784).

The present study, however, went beyond the concept of resistance to change to consider the concept of positive change, namely the acceptance of change (AC), in line with recent advances in positive psychology (Seligman and Csikszentmihalyi, 2000; Compton, 2005) and bearing in mind Maslow's theory of needs (1954) and evolutionary theory (Maslow, 1954). To quote Zuckerman (2014), “change is a fact of life, that people can resist or accept, considering that the world around us inevitably will change” (Zuckerman, 2014, p. 1). Based on this premise, the ability to accept change could be a very useful resource in many spheres of life at the intra- and inter-individual level as well as the global level.

A new construct, up till now missing in the psychology literature, is therefore needed to study the phenomenon of change. This new construct is the acceptance of change (AC), and researchers are currently engaged in verifying the main psychometric properties of a new multidimensional scale for assessing AC in its different forms.

## Analysis of different constructs in relation to AC

A review of the literature on constructs that could have a bearing on AC will help explain why we defined a new construct and developed a new measure for its evaluation. In particular, we would like to focus on research on the process of change in the work and organizational fields on the basis of different variables seemingly contributing to change.

### The concept of resistance to change (RC)

The first studies on resistance to change in work and organizational psychology were published in the late 1940s and early 1950s (Coch and French, 1948). Later studies analyzed resistance to change as a defense mechanism (Zander, 1950) and its social impact as such a mechanism (Lawrence, 1954). Early research on change focused primarily on how organizations as a whole reacted to organizational change (Armenakis and Bedeian, 1999) and not on individuals' reaction or attitude to such change (Vakola et al., 2004).

More recent studies have begun to explore resistance to change variables from an individual perspective (Judge et al., 1999; Piderit, 2000; Oreg, 2003; Lamm and Gordon, 2010) although problems have emerged in the analysis of this construct (Dent and Goldberg, 1999; Piderit, 2000). Dent and Goldberg (1999) point out that previous studies concentrated on ways of preventing resistance to change phenomena, while Piderit (2000) states that earlier researchers considered resistance to change a behavioral, emotional, or cognitive reaction and proposed an integrated model for such resistance. Oreg (2003) defines resistance to change as the individual tendency to resist or avoid change, to devalue change in general, and to label it as negative regardless of the context (Oreg, 2003). All these studies investigated individuals' attitudes in terms of resistance to change. The present study, by contrast, explored and conceptualized a new construct, acceptance of change (AC), and developed a new measure for assessing it.

### The concept of openness to change

Openness to change is a construct introduced by Wanberg and Banas (2000) based on cognitive adaptation theory, which holds that individuals with the highest levels of well-being during stressful life events are those who also have high levels of self-esteem, optimism, and perceived control (Wanberg and Banas, 2000). Several authors have pointed out also the usefulness of psychological resources in building a resilient personality (Major et al., 1998) and being open to change (Taylor and Brown, 1988; Judge et al., 1997).

In describing the construct of openness to change in a reorganizing workplace, Wanberg and Banas (2000) list several variables including receipt of information on the change, participation in the change process, self-efficacy in the belief to be able to change, social support, and the personal impact of the change. According to these authors, the mentioned variables are often more responsive to organizational intervention than the other variables in cognitive adaptation theory (self-esteem, generalized perceived control, and optimism) (Wanberg and Banas, 2000).

## The acceptance of change (AC): A new construct

The literature reveals major consensus regarding the role of human nature in resistance to change (Coch and French, 1948; Zander, 1950; Conner, 1992; Oreg, 2003). Some authors, however, consider resistance to change counterproductive (Dent and Goldberg, 1999).

Acceptance of change (AC) can be seen as the tendency to embrace rather than shy away from change because acceptance is regarded as positive for a person's well-being. AC thus stems from the belief that, in their work and other activities, people who are able to accept change often find that the change has a positive impact on their working lives and their resource levels. In terms of Maslow's theory of needs (1954) and evolutionary theory, AC can be conceptualized as a multidimensional, dynamic construct, a core of positive characteristics that can be improved and acted on preventively (Maslow, 1954). In terms of positive psychology AC, can be viewed as a chance to grow and learn (Seligman and Csikszentmihalyi, 2000; Wanberg and Banas, 2000).

In defining this new construct and its dimensions, we relied on the above conceptualization and, in particular, on studies on change in the work and organizational fields, analyzing the variables seemingly contributing to change (e.g., Goldsmith, 1984; Mallinckrodt and Fretz, 1988; Lau and Woodman, 1995; Oreg, 2003; Vakola et al., 2004; Jordan, 2005; Visser, 2010). Given that the traditional approach involved investigating individuals' attitudes in terms of resistance to change (Oreg, 2003), we considered it appropriate in this study to investigate these attitudes in terms of positive characteristics and dimensions for implementing change. The identified dimensions were: Predisposition to Change; Support for Change; Change Seeking; Positive Reaction to Change; and Cognitive Flexibility.

### Predisposition to change

Predisposition to change is the ability people have to learn from change and to use change to improve the quality of their lives. Self-determination theory holds that the drive for change can be intrinsically and extrinsically motivated (Deci and Ryan, 1985, 2000) and that people will be supported by their natural or intrinsic tendencies to behave in effective and healthy ways and to cope with change. Gagné and Deci (2005) found a link between motivation and several organizational variables including persisting with and maintaining behavior change (Visser, 2010).

### Support for change

Support for change refers to social support perceived to be received from others when facing challenges. Many researchers stress the importance of social support in promoting well-being and life satisfaction (Mallinckrodt and Fretz, 1988; Shaw et al., 1993). Perceived social support refers to people's perception that they are receiving help and support from others, for example the support of parents and friends perceived to be received, particularly emotional support (reassurance, encouragment) and cognitive support (advice, suggestions, comparisons).

### Change seeking

The process of change frequently leads to an increase in mental and physiological stimulation in people. Zuckerman (2014) argues that some individuals seek change and that this could have adaptive value for those who are able to acquire and retain information. According to Maslow's theory of needs (1954)—which stresses the importance of growth, change, and openness to new experiences—people are driven toward change by their own needs. Moreover, several studies have found that innovative individuals tend to find new solutions to problems outside the normal framework of problem solving (Kirton, 1980, 1989) and often exhibit a need for new stimuli (Goldsmith, 1984; Oreg, 2003).

Change seeking appears to be a key factor in the acceptance of change (AC) process because individuals with a high level of AC generally have a high level of novelty seeking. Change seeking predisposes individuals not only to seek change, but also to accept and integrate life and work changes not necessarily originating from them.

### Positive reaction to change

The broaden-and-build theory (Fredrickson, 2001, 2004) holds that positive emotions broaden people's awareness and encourage them to try new things. According to Fredrickson (2001, 2004), such positive emotions incline people toward new possibilities and enable them to make good choices. This theory partly inspired the present acceptance of change (AC) construct—and particularly the positive reaction to change (PRC) dimension—because positive emotions predispose people to experience change positively and to benefit from it.

Jordan (2005) argues that change is inherently emotional and that there is, accordingly, a theoretical justification for the notion that people with greater emotional intelligence tend to be more efficient in managing change compared to those with lower emotional intelligence. Vakola et al. (2004) claim also that emotional intelligence can contribute to organizational change and that it is therefore a critical success factor in the change process (Di Fabio and Palazzeschi, 2009; Di Fabio et al., 2016). The PRC factor thus plays a key role in the acceptance of change.

### Cognitive flexibility

Cognitive flexibility (CF) can be defined as the mental ability to switch between different concepts (Scott, 1962) or to adapt cognitive processing strategies (Cañas et al., 2005).

Cognitive reaction to change refers to what people think about change, and cognitive flexibility (CF) refers to people's ability to think about multiple concepts simultaneously, to change decisions if this is advantageous, and to change plans and routines easily. Little is known about CF in relation to change because, in the past, researchers focused primarily on rigidity and close-mindedness, which are prominent factors in people's resistance to change (Lau and Woodman, 1995; Oreg, 2003).

## Method

### Participants and procedure

There were 261 participants in the present study (148 men, 103 women) with a mean age of 37.36 years old (*SD* = 17.74). The sample consisted of 141 workers (124 men) and 120 students (96 women) of the University of Florence.

The participants completed all the instruments discussed in the Measures Section (below), which were administered in line with privacy norms. The participants' anonymity was guaranteed, and written informed consent was obtained from them after a full description of the study. The participants were told they could leave the study at any time and that there would be no payment for participating. The study adhered to the latest version of the Declaration of Helsinki revised in Fortaleza [World Medical Association (WMA), 2013] with regard to ethical standards for research.

### Measures

#### Acceptance of change scale (ACS; Di Fabio and Gori, in review)

The Acceptance of Change Scale (ACS) is a self-report measure that assesses the tendency of clients to accept or move toward change. The dimensions of the measure are Positive Reaction to Change, Change Seeking, Cognitive Flexibility, Predisposition to Change, and Support for Change. The ACS uses a 5-point Likert-type scale (1 = *not at all*, 2 = *a little*, 3 = *somewhat*, 4 = *much*, 5 = *a great deal*) that consists of 20 items (e.g., “When I am faced with a change, I can see things from multiple perspectives,” “I normally seek different ways to do the same things in my daily routine,” “I am able to give new meanings to the things that I have been doing for a long time,” “It's easy for me to change my mind when I realize that I am wrong”). This scale can be used in the work and organizational fields and in all situations where it is necessary to promote change (e.g., human resources, counseling, empowerment) in order to help people understand themselves better and find their own way to manage their life and work opportunities.

#### Intrapreneurial self-capital scale (ISCS; Di Fabio, 2014a)

Intrapreneurial self-capital is defined as a core of individual intrapreneurial resources used to cope with career and life construction challenges and includes dimensions of core self-evaluation, hardiness, creative self-efficacy, resilience, goal mastery, decisiveness, and vigilance (Di Fabio, 2014a). The Intrapreneurial Self-Capital Scale was developed by Di Fabio (2014a) to measure this new construct. The ISCS uses a 5-point Likert-type scale (1 = *strongly disagree*, 2 = *disagree*, 3 = *neither agree nor disagree*, 4 = *agree*, 5 = *strongly agree*) that consists of 28 items (e.g., “I am able to deal with most of my problems,” “I am able to improve the ideas produced by others,” “I am able to achieve objectives despite obstacles,” “One of my goals in training is to learn as much as I can”). In the present study, we used the Italian version of this measure, which showed good internal consistency (*a* = 0.86) (Di Fabio, 2014a).

#### Psychological self-capital scale (PS; Luthans et al., 2007)

Psychological capital is a new construct measured with the Psychological Capital Questionnaire 24 (PCQ-24). This measure of 24 items has four subscales: (1) Efficacy/Confidence (e.g., “I feel confident contributing to discussions about the organization's management”), (2) Hope (e.g., “I can think of many ways to reach my current work goals”), (3) Optimism (e.g., “I'm optimistic about what will happen to me in the future regarding work”), and (4) Resilience (e.g., “I usually take stressful things at work in my stride”). Each of these subscales consists of six items with response options on a six-point Likert scale ranging from 1 (*strongly disagree*) to 6 (*strongly agree*). The PCQ-24 has good psychometric properties, also in its Italian version, which was used in the present study (hope = 0.75; efficacy = 0.78; resilience = 0.70; optimism = 0.77; the overall scale = 0.81) (Alessandri et al., 2015).

#### Flourishing scale (FS; Diener et al., 2010)

The Flourishing Scale (FS) is a self-report measure consisting of eight items that assess respondents' self-perceived success in important areas of their lives such as relationships, self-esteem, purpose, and optimism. The response options are on a 6-point Likert scale ranging from 1 (*strongly disagree*) to 7 (*strongly agree*). Examples of items are “My social relationships are supportive and rewarding,” “I lead a purposeful and meaningful life,” “I am optimistic about my future.” The FS showed a unidimensional structure with good reliability (Diener et al., 2010), and the Italian version of the scale (Di Fabio, 2016a) also demonstrated good reliability (*a* = 0.88).

#### Positive relational management scale (PRMS; Di Fabio, 2015b)

The Positive Relational Management Scale (PRMS) is a measure consisting of 12 items on a 5-point Likert scale ranging from 1 (*strongly disagree*) to 5 (*strongly agree*). The PRMS is used to assess three dimensions (respect, caring, connection) and has a total score (e.g., Respect: “I keep a balance between respect toward others and toward myself”; Caring: “I often take care of others”; Connection: “I have good relationships with my family”). The psychometric properties of the scale are good, also in its Italian version (respect = 0.81; caring = 0.79; connection = 0.80; PRMS total = 0.84) (Di Fabio, 2015b).

#### Satisfaction with life scale (SWLS; Diener et al., 1985)

The Satisfaction With Life Scale (SWLS) is a self-report instrument that measures global life satisfaction. It consists of five items on a seven-point Likert scale with higher values corresponding to higher degrees of life satisfaction. Examples of the items are “I am satisfied with my life,” “The conditions of my life are excellent.” The psychometric properties of the SWLS are good, and different studies have reported a unidimensional structure for this instrument (Diener et al., 1985; Vera-Villarroel et al., 2012). The Italian version of the SWLS (*a* = 0.85) was used in the study (Di Fabio and Gori, 2015).

#### Multidimensional scale of perceived social support (MSPSS; Zimet et al., 1988)

The MSPSS consists of 12 items on a 7-point Likert-type scale ranging from 1 = *strongly disagree* to 7 = *strongly agree*. It measures perceived support related to three main domains: family, friends, and a significant other. Examples of the items are “My family works very hard to help me,” “I can speak about my problems with my friends,” “When I need someone, there is always a special person who stands by me.” Higher scores suggest greater levels of perceived social support. The MSPSS has been found to have good psychometric properties in several studies with different samples (Zimet et al., 1988, 1990). The Italian version of the scale was used in this study and reported excellent internal consistency for the three factors: family (α = 0.91), friends (α = 0.93), and significant other (α = 0.88), and the total score (α = 0.92) (Di Fabio and Palazzeschi, 2015b).

#### Ten item personality inventory (TIPI; Gosling et al., 2003)

The Ten Item Personality Inventory (TIPI) was developed using descriptors related to the Big Five measures with each item consisting of two adjectives, separated by a comma, rated on a 7-point likert scale ranging from 1 (*strongly disagree*) to 7 (*strongly agree*). TIPI assesses the following dimensions linked to the Big Five measures: Extraversion (E) (e.g., “I see myself as extraverted, enthusiastic”), Agreeableness (A) (e.g., “I see myself as sympathetic, warm”), Conscientiousness (C) (e.g., “I see myself as dependable, self-disciplined”), Neuroticism (N) (e.g., “I see myself as calm, emotionally stable”), Openness (O) (e.g., “I see myself open to new experiences, complex”). The Italian version of this brief measure was used in the study and showed good values of internal reliability (E = 0.82; A = 0.78; C = 0.79; N = 0.71; O = 0.74) (Di Fabio et al., 2016).

#### Rosenberg self-esteem scale (RSES; Rosenberg, 1965)

The Rosenberg Self-Esteem Scale (RSES) is a ten-item scale for assessing global self-esteem with items answered on a 4-point Likert scale ranging from *strongly agree* to *strongly disagree* (e.g., “On the whole, I am satisfied with myself,” “I have a positive attitude toward myself.”). The psychometric properties of the RSES as reported in several studies are good (Corwyn, 2000). The Italian version of the RSES (*a* = 0.84) was used in the present study (Prezza et al., 1997).

#### Insight orientation scale (IOS; Gori et al., 2015)

The Insight Orientation Scale (IOS) is a brief measure consisting of seven items that assess clients' insight capacity, their behaviors, and their feelings and opinions about this construct (Gori et al., 2015). The response format is a 5-point Likert scale (1 = “*not at all*;” 2 = “*a little*;” 3 = “*somewhat*;” 4 = “*a lot*,” and 5 = “*a great deal*”) (e.g., “I am aware of the things I am doing,” “I am able to solve difficult problems”). The IOS showed good psychometric properties (*a* = 0.77) (Gori et al., 2015).

#### Resistance to change scale (RCS; Oreg, 2003)

The Resistance to Change Scale (RCS) is a measure consisting of 21 items that are responded to on a 5-point Likert-type scale ranging from 1 = *absolutely disagree* to 5 = *absolutely agree* (e.g., “Changing plans seems like a real hassle to me,” “I like to do the same old things rather than try new and different ones”). The measure identifies three factors: routine seeking, emotional reaction to imposed change, and cognitive rigidity. The Cronbach's alphas in the study were 0.70 for routine seeking, 0.67 for emotional reaction to imposed change, and 0.66 for cognitive rigidity. The Italian version of the RCS (Di Fabio and Bernaud, 2007) was used in the study.

#### Authenticity scale (AS; Wood et al., 2008)

The Authenticity Scale (AS) is a measure consisting of 12 items with a response format on a 7-point Likert scale ranging from 1 = *does not describe me at all* to 7 = *describes me very well*. The measure assesses Authenticity in terms of three dimensions (self-alienation, authentic living, and accepting external influence) and a total score (e.g., “I am true to myself in most situations”). The Italian version of AS (Di Fabio, 2014b) was used in the study and showed good psychometric properties (*a* = 0.77) (Di Fabio, 2014b).

### Data analysis

Factor analysis was used to identify the underlying dimensions of the ACS, using various criteria for item selection according to the number of selected factors and item factor loadings. In order to verify some assumptions, prior to exploratory factor analysis (EFA), we analyzed the Bartlett's Test of Sphericity and the Kaiser-Meyer-Olkin's (KMO) Measure of Sampling Adequacy to assess if the items were significantly correlated and shared sufficient variance to justify factor extraction. Principal axis factoring was selected as the method of factor extraction with oblique rotation (promax criterion) to obtain a simple structure as there was no theoretical assumption to suggest that the factors were independent of each other. Eigenvalues >1, the Kaiser criterion, and the screen test were checked for agreement (Giannini et al., 2011), and their salience was determined by applying the following criteria: (a) a factor loading of at least 0.3 on the primary factor, ensuring a high degree of association between the item and the factor; (b) a difference of 0.3 between loading on the primary factor and loading on other factors, ensuring that each item could be considered salient to one factor when an item was loading simultaneously on two factors; (c) a minimum of three items for each factor thus ensuring meaningful interpretation of stable factors (Craparo et al., 2015). The standard Pearson correlation coefficient was used to investigate to what extent the factor scores were intercorrelated. The reliability of the scale, in terms of internal consistency, was calculated by means of the alpha coefficient. A confirmatory factor analysis (CFA) was performed using maximum likelihood (ML) estimation procedures. To assess the closeness of the hypothetical model to the empirical data statistically, multiple goodness-of-fit indexes were used, including the ratio of the chi-square to degrees of freedom (χ2/*df*), the Tucker-Lewis Index (TLI), the Comparative Fit Index (CFI), the Standardized Root Mean Square Residual (SRMR), and the Root Mean Square Error of Approximation (RMSEA). Bentler and Bonnet (1980) contend that values >0.90 indicate good fit for the TLI (Hu and Bentler, 1999) contend that CFI values >0.90 are needed; and Byrne (1994) contends that a cutoff of 0.93 should indicate a good fit. SRMR and RMSEA values < 0.08 (Browne and Cudeck, 1993), and ideally equal to or < 0.05, are interpreted as indicating models that fit well (Steiger, 1990; Schermelleh-Engel et al., 2003; Giannini et al., 2011). Several aspects of concurrent validity were verified using the Pearson's *r* coefficient.

## Results

An examination of the scree plot (Cattell, 1966), and percentage of variance accounted for, revealed that as many as five factors should be retained for rotation. The exploratory factor analysis (EFA) (promax rotation) showed a factor structure with five principal dimensions (eigenvalues >1; 6.52, 2.09, 1.47, 1.33, 1.23), with 63.21% of total variance explained.

Factor 1 (Predisposition to Change), with four items loading above 0.56, had an eigenvalue of 6.52 and accounted for 32.58% of the total variance explained. Factor 2 (Support for Change), with four items loading above 0.41, had an eigenvalue of 2.09 and accounted for 10.46% of the total variance explained. Factor 3 (Change Seeking), with four items loading above 0.54, had an eigenvalue of 1.47 and accounted for 7.36% of the total variance explained. Factor 4 (Positive Reaction to Change), with four items loading above 0.40, had an eigenvalue of 1.33 and accounted for 6.65% of the total variance explained. Factor 5 (Cognitive Flexibility), with four items loading above 0.46, had an eigenvalue of 1.23 and accounted for 6.15% of the total variance explained. The factor structure matrix shows the five independent factors of the questionnaire (Table 1). The revealed dimensions correlated significantly and showed good values (from *r* = 0.207, *p* < 0.01, to *r* = 0.510, *p* < 0.01) (Table 2).

**Table 1:** **Factor structure matrix of the Acceptance of Change Scale (ACS)**.

	**Predisposition to change**	**Support for change**	**Change seeking**	**Positive reaction to change**	**Cognitive flexibility**
10. Thinking about new plans is easy for me *Pensare a nuovi piani è facile per me*	0.904				
9. I easliy identify alternative paths *Individuo percorsi alternativi con facilità*	0.891				
11. When I am faced with a change, I can see things from multiple perspectives *Di fronte ad un cambiamento so vedere le cose da molteplici prospettive*	0.726				
12. I am able to take all the opportunities that occur to me *Sono in grado di afferrare tutte le opportunità che mi si presentano*	0.560				
18. I trust the people close to me when faced with change *Mi fido delle persone a me vicine di fronte ai cambiamenti*		0.862			
19. When I compare myself with others, I am better able to cope with change *Quando mi confronto con gli altri riesco ad affrontare meglio i cambiamenti*		0.683			
17. I can compare myself with other people important to me when facing change *Posso confrontarmi con altre persone per me importanti di fronte ai cambiamenti*		0.669			
20. I can handle the changes in relationships with others *Riesco a gestire i cambiamenti nei rapporti con gli altri*		0.415			
4. Although I do not see the benefits, I cannot wait to change *Anche se non vedo benefici, non vedo l'ora di cambiare*			0.867		
3. I am looking for changes in my life, even when things are going well *Sono in cerca di cambiamenti nella mia vita, anche quando le cose vanno bene*			0.746		
1. I am always looking for changes in my everyday life *Sono sempre in cerca di cambiamenti nella mia vita di tutti i giorni*			0.597		
2. I normally seek different ways to do the same things in my daily routine *Normalmente cerco modi diversi per fare le stesse cose della mia routine giornaliera*			0.541		
15. I am able to give new meanings to the things that I have been doing for a long time *Sono in grado di dare nuovi significati alle cose che faccio da lungo tempo*				0.824	
16. I am aware of mutations that involve the change *Sono consapevole delle modifiche che comporta il cambiamento*				0.675	
14. I am able to tolerate even the negative aspects of change *Sono in grado di tollerare anche gli aspetti negativi del cambiamento*				0.566	
13 I can find the positives in changes that are apparently negative *Riesco a trovare lati positivi nei cambiamenti che sono apparentemente negativi*				0.405	
6. My opinions may have changed *Le mie opinioni possono cambiare*					0.771
5. If necessary, it is not difficult for me to change my mind *Se necessario, non è difficile per me cambiare idea*					0.628
8. When I've made an important decision, I can change it if it involves an advantage *Quando ho preso una decisione importante posso cambiarla, se questo comporta un vantaggio*					0.513
7. It's easy for me to change my mind when I realize that I am wrong *E' facile per me cambiare idea quando mi rendo conto che sono in errore*					0.459
% explained variance	32.58%	10.46%	7.36%	6.65%	6.15%

^*^Italian version of the scale.

**Table 2:** **Intercorrelations of the ACS dimensions**.

	**1**	**2**	**3**	**4**
1. Predisposition to change	1			
2. Support for change	0.469^**^	1		
3. Change seeking	0.440^**^	0.207^**^	1	
4. Positive reaction to change	0.510^**^	0.449^**^	0.406^**^	1
5. Cognitive flexibility	0.475^**^	0.317^**^	0.375^**^	0.455^**^

** *p < 0.01*.

The goodness-of-fit indices showed a good fit of the model to the data. Although the chi-square was significant, the other goodness-of-fit indices showed satisfactory and good values (*x*^2^/df = 1.81 *p* < 0.001; CFI = 0.94; TLI = 0.92; SRMR = 0.05; RMSEA = 0.05).

The reliability of the scales, calculated using the Cronbach's alpha coefficient, indicated good values of internal consistency (Predisposition to Change, *a* = 0.83; Support for Change, *a* = 0.79; Change Seeking, *a* = 0.80; Positive Reaction to Change, *a* = 0.75; Cognitive Flexibility, *a* = 0.72, and the overall scale = 0.88).

The ACS showed strong correlations with the measures used to assess some aspects of concurrent validity (Tables 3, 4).

**Table 3:** **Summary of the correlation between the ACS and ISC, PSC, F, IOS, PSR, MSPSS**.

	**ISCS**	**PSCS**	**FS**	**IOS**	***PRMS***	***MSPSS***
1. Predisposition to change	0.550^**^	0.611^**^	0.428^**^	0.437^**^	0.401^**^	0.309^**^
2. Support for change	0.425^**^	0.361^**^	0.354^**^	0.274^**^	0.406^**^	0.458^**^
3. Change seeking	0.375^**^	0.360^**^	0.208^**^	0.328^**^	0.199^**^	0.094
4. Positive reaction to change	0.440^**^	0.463^**^	0.332^**^	0.434^**^	0.301^**^	0.119
5. Cognitive flexibility	0.357^**^	0.405^**^	0.179^**^	0.376^**^	0.229^**^	0.117
AC total score	0.591^**^	0.604^**^	0.411^**^	0.507^**^	0.420^**^	0.300^**^

** *p < 0.01*.

*ISCS, Intrapreneurial Self-Capital Scale; PSCS, Psychological Self-Capital Scale; FS, Flourishing Scale; IOS, Insight Orientation Scale; PRMS, Positive Relational Management Scale; MSPSS, Multidimensional Scale of Perceived Social Support*.

**Table 4:** **Summary of the correlation between the two parts of the ACS and I-TIPI, RSES, SWLS, AS, RCS**.

	**TIPI E**	**TIPI A**	**TIPI C**	**TIPI ES**	**TIPI O**	**RSES**	**SWLS**	**AS**	**RCS**
1. Predisposition to change	0.290^**^	0.295^**^	0.254^**^	0.392^**^	0.438^**^	0.433^**^	0.432^**^	193^**^	−0.431^**^
2. Support for change	0.218^**^	0.330^**^	0.199^**^	0.265^**^	0.162^**^	0.357^**^	0.465^**^	0.169^**^	−0.222^**^
3. Change seeking	0.192^**^	0.121	0.117	0.223^**^	0.392^**^	0.110	0.136^*^	−0.10	−0.410^**^
4. Positive reaction to change	0.134^*^	0.162^**^	0.168^**^	0.287^**^	0.301^**^	0.240^**^	0.287^**^	0.168^**^	−0.335^**^
5. Cognitive flexibility	0.080	0.256^**^	0.171^**^	0.193^**^	0.240^**^	0.174^**^	0.151^**^	−0.008	−0.262^**^
AC total score	0.255^**^	0.318^**^	0.248^**^	0.374^**^	0.428^**^	0.358^**^	0.401^**^	0.136^*^	−0.463^**^

** p < 0.01,

* *p < 0.05*.

*TIPI, Ten Item Personality Inventory; RSES, Rosenberg Self-Esteem Scale; SWLS, Satisfaction With Life Scale; AS, Authenticity Scale; RCS, Resistance to Change Scale*.

## Discussion

People in the twenty-first century need the skills and resources to manage uncertainty and to face change in the most adaptive way (Arnoux-Nicolas et al., 2016; Di Fabio and Maree, 2016). This is even more important where the degree of uncertainty is high, for example at work. Work and organizational psychologists tend to see change as a process of gradual adaptation when, for example, individuals in organizations react to internal and external pressures (Demers, 2007) or when change results from selections made in terms of evolutionary theory (Child and Kieser, 1981; Hrebiniak and Joyce, 1985; Demers, 2007; Dunican, 2015). According to Topping (2002), change implies uncertainty, and uncertainty can cause fear. However, acknowledging uncertainty can lead to the acceptance of challenges (Geller, 2002; Dunican, 2015). Some researchers place more emphasis on the predisposition of people to accept or resist change (Michel et al., 2013) than on other factors such as leadership or the management of functional boundaries (Huczynski and Buchanan, 2001; Hoag et al., 2002; Burnes, 2003).

Acceptance of change as an individual reaction offers opportunities for future research as acceptance of change as opposed to resistance to change can promote people's effective and lifelong self and relational management (Di Fabio, 2015b; Di Fabio and Kenny, 2016). In other words, while recognizing the importance of the Oreg's (2003) resistance to change construct, in this study we wanted to turn the focus from negative to positive in order to emphasize the preventive perspective in psychology (Hage et al., 2007; Kenny and Hage, 2009; Di Fabio, 2015a; Di Fabio and Kenny, 2016) and to underline the importance of positive psychology (Seligman and Csikszentmihalyi, 2000).

The study accordingly set out to develop a new measure for assessing acceptance of change and its psychometric properties. The EFA showed a good structure with five principal dimensions: Predisposition to Change; Support for Change; Change Seeking; Positive Reaction to Change; and Cognitive Flexibility. Nunnally and Bernstein (1994) found that these dimensions showed good reliability; in fact, despite the reduced number of items, each dimension presented a good Cronbach's alpha.

The CFA also revealed a good fit of the model to the empirical data, as indicated by the fit indices. In particular, the CFA results showed that the SRMS and RMSEA were equal to 0.05, indicating a good fit (Browne and Cudeck, 1993; MacCallum et al., 1996). The CFI index indicated a good fit too (Byrne, 1994) and the TLI index a satisfactory fit (Bentler and Bonnet, 1980).

Correlations between the ACS and the measures used to verify some aspects of concurrent validity showed good values: all the relationships between the variables under investigation were in the right direction and with the right significance. In particular, the relationships between the ACS and these measures indicated that change is strictly related to individual intrapreneurial resources (Intrapreneurial Self-Capital Scale), to self-capital (Psychological Self-Capital Scale), to insight capacity (Insight Orientation Scale), to self-perceived success (Flourishing Scale), to life satisfaction (Satisfaction With Life Scale), and to openness (TIPI-Openness). Acceptance of change is thus particularly important in individual resource construction. Further studies are needed to explore these relationships in greater depth.

Also, the strong, negative correlation between the ACS and the Resistance of Change Scale (RCS) indicated the good divergent validity of the ACS.

Regarding the limitations of the present study, the major limitation was the relatively small number of participants. The use of so many instruments also burdened the administration process. However, at the same time, the large number of scales used in the study to assess aspects of concurrent validity could be viewed as a strength because it enabled the comparison of many different constructs thus providing additional information. Future studies on the topic should make use of larger samples with strict selection of the constructs to be investigated.

On the positive side, the study showed that the ACS is a brief and easily administered instrument with good psychometric properties that can be used to help people adjust to change and find their own way to manage their life and work opportunities.

Because resistance to change can be seen as a counterproductive attitude (Dent and Goldberg, 1999), we preferred to focus on acceptance of change as a productive attitude. In fact, the acceptance of change can move people from a state of immobility to a state of mobility and thereby help them meet the challenges of the twenty-first century (Di Fabio and Bernaud, 2008; Di Fabio and Maree, 2013; Di Fabio, 2015b, 2016b; Di Fabio et al., 2015) and achieve general well-being (Di Fabio and Kenny, 2012a,b, 2015, 2016; Di Fabio and Maree, 2012; Di Fabio and Bucci, 2016; Di Fabio and Palazzeschi, 2016). In other words, the ability to accept change can be a valuable resource in many spheres of life on an intra- and inter-individual level as well as a global level, and it is hoped that the ACS will prove to be a useful tool in this endeavor.

## Author contributions

AD conceptualized the study and choose the theoretical framework. AD conceptualized the new scale and realized it. AD and AG collected the data. AG analyzed the data and wrote the methods and results. Then all authors wrote the paper together and read and revised the manuscript several times.

### Conflict of interest statement

The authors declare that the research was conducted in the absence of any commercial or financial relationships that could be construed as a potential conflict of interest.
